# An investigation of conventional microbial culture for the *Naja atra* bite wound, and the comparison between culture-based 16S Sanger sequencing and 16S metagenomics of the snake oropharyngeal bacterial microbiota

**DOI:** 10.1371/journal.pntd.0009331

**Published:** 2021-04-15

**Authors:** Yan-Chiao Mao, Han-Ni Chuang, Chien-Hung Shih, Han-Hsueh Hsieh, Yu-Han Jiang, Liao-Chun Chiang, Wen-Loung Lin, Tzu-Hung Hsiao, Po-Yu Liu

**Affiliations:** 1 Department of Emergency Medicine, Division of Clinical Toxicology, Taichung Veterans General Hospital, Taichung, Taiwan; 2 School of Medicine, National Defense Medical Center, Taipei, Taiwan; 3 Department of Medicine, Division of Clinical Toxicology and Occupational Medicine, Taipei Veterans General Hospital, Taipei, Taiwan; 4 Institute of Environmental and Occupational Health Sciences, School of Medicine, National Yang Ming Chiao Tung University, Taipei, Taiwan; 5 Department of Medical Research, Taichung Veterans General Hospital, Taichung, Taiwan; 6 Precision Medicine Center, Taichung Veterans General Hospital, Taichung, Taiwan; 7 National Tsing Hua University, College of Life Sciences, Hsinchu, Taiwan; 8 Taichung Wildlife Conservation Group, Taichung, Taiwan; 9 Department of Public Health, Fu Jen Catholic University, New Taipei City, Taiwan; 10 Institute of Genomics and Bioinformatics, National Chung Hsing University, Taichung, Taiwan; 11 Department of Internal Medicine, Division of Infectious Diseases, Taichung Veterans General Hospital, Taichung, Taiwan; 12 Rong Hsing Research Center for Translational Medicine, National Chung Hsing University, Taichung, Taiwan; 13 Ph.D. Program in Translational Medicine, National Chung Hsing University, Taichung, Taiwan; Institut de Recherche pour le Développement, BENIN

## Abstract

*Naja atra* is a major venomous snake found in Taiwan. The bite of this snake causes extensive wound necrosis or necrotizing soft tissue infection. Conventional microbial culture-based techniques may fail to identify potential human pathogens and render antibiotics ineffective in the management of wound infection. Therefore, we evaluated 16S Sanger sequencing and next-generation sequencing (NGS) to identify bacterial species in the oropharynx of *N*. *atra*. Using conventional microbial culture methods and the VITEK 2 system, we isolated nine species from snakebite wounds. On the basis of the 16S Sanger sequencing of bacterial clones from agar plates, we identified 18 bacterial species in the oropharynx of *N*. *atra*, including *Morganella morganii*, *Proteus vulgaris*, and *Proteus mirabilis*, which were also present in the infected bite wound. Using NGS of 16S metagenomics, we uncovered more than 286 bacterial species in the oropharynx of *N*. *atra*. In addition, the bacterial species identified using 16S Sanger sequencing accounted for only 2% of those identified through NGS of 16S metagenomics. The bacterial microbiota of the oropharynx of *N*. *atra* were modeled better using NGS of 16S metagenomics compared to microbial culture-based techniques. *Stenotrophomonas maltophilia*, *Acinetobacter baumannii*, and *Proteus penneri* were also identified in the NGS of 16S metagenomics. Understanding the bacterial microbiota that are native to the oropharynx of *N*. *atra*, in addition to the bite wound, may have additional therapeutic implications regarding empiric antibiotic selection for managing *N*. *atra* bites.

## Author summary

*Naja atra* bites induce extensive wound necrotizing soft tissue infections in a substantial proportion of patients. Empiric antibiotic administration in snakebite patients is a common practice, but clinical reports indicate that this treatment was ineffective in preventing secondary infection given that the microbiota of the infected wound and oropharynx of the culprit snake were not properly established. In this study, only 9 species were detected in cobra bites using a *conventional microbial culture* method and the VITEK 2 system, whereas 18 species were detected in the cobra oropharynx using *microbial culture*-based 16S Sanger sequencing. Among these, *Morganella morganii*, *Proteus vulgaris*, and *Proteus mirabilis* were identified as common bacteria. Compared to *microbial culture*-based 16S Sanger sequencing, NGS-based 16S metagenomic sequencing detected more than 286 bacterial species. *Stenotrophomonas maltophilia*, *Acinetobacter baumannii*, *and Proteus penneri* only appeared with 16S metagenomic sequencing. These results suggest that NGS-based 16S metagenomic sequencing is a better tool for uncovering the bacterial microbiota of the N. atra oropharynx, which may help in developing a proper therapeutic strategy for patients with *N*. *atra* bites.

## Introduction

Snakebites are one of the most neglected tropical diseases [1, 2]. Worldwide, more than five million people are bitten and up to 2.7 million are envenomed every year [1]. Wound infection is a severe complication after a snakebite and is associated with considerable mortality and morbidity [3–6]. Among common venomous snakes in Asian regions, *Naja* species with cytotoxic venoms carry the highest risk of wound infection [7]. *N*. *atra* is the only *Naja* species distributed in Taiwan. This snake accounts for only 6% of all snake bite incidents but causes the most severe infectious complications among the major venomous snakes [7, 8]. Furthermore, wound necrosis or necrotizing soft tissue infections are frequently reported [5, 9, 10]. Using conventional culturing techniques [11], *Enterococcus* spp. and *Morganella morganii* were the most commonly isolated pathogens in infected *N*. *atra* bite wounds, which is similar to the snake’s oral cavity [5, 7, 12, 13]. However, in spite of the aggressive administration of specific antivenom, antibiotics, or both, more than 50% of patients underwent surgery because of wound infection following a bite [5]. The high surgical proportion may be due to the poor efficacy of the antivenom against the cytotoxic components of the venom, failure of conventional culture-based techniques to report less significant or nonculturable microorganisms, including potential human pathogens, or improperly chosen empiric antibiotics [14–16].

Patients with snakebites receive several forms of treatment (e.g., wound cleansing, topical herb application, or prophylactic antibiotic administration) that might change the bacterial composition and loads recovered from cultures [7]. Although empiric antibiotic administration in patients with snake bites is a common practice [17], it may be ineffective in preventing secondary infection if the microbiology of the bite wound and oropharynx of the culprit snake is not comprehensively determined [10]. Therefore, we first glance at the features of infected *N*. *atra* bite patients and the bacteria recovered from the bite wound based on a conventional microbial culture method in clinical settings. We then compare the capacity of conventional microbial culture-based 16S Sanger sequencing and next-generation sequencing (NGS) methods to evaluate the bacterial microbiota of the oropharynx of *N*. *atra* and to better appraise microbial diversity and therapeutic implications.

## Materials and methods

### Ethics statement

The human study was approved by the Institutional Review Board (CE16225A) and the snake study was conformed to the National Law.

### Patient information

All *N*. *atra* bite cases were admitted to Taichung Veterans General Hospital between January 2001 and July 2006. Patients with suspected wound infections who received a deep tissue or biopsy microbial culture were carefully examined. The following data were collected: age, sex, bitten body part, clinical manifestations, and management (including the dosage and timing of specific antivenom administration, indication and timing for surgery, and types of surgery). We used the same criteria as Mao et al. [5, 7] to define wound infection and other complications. Some study participants overlapped with previous studies that had different reference periods [5,7].

Deep tissue or biopsy microbial cultures (aerobic and anaerobic) were performed during surgical debridement. Bacteria were identified using the VITEK 2 system (BioMérieux Inc., Durham, NC, USA). Culture sampling was performed as previously described [11].

### Sample collection and identification of isolated bacteria in the *N*. *atra* oropharynx

All cobras in this study were collected in 2020 from various locations in central Taiwan by a snake rescue team. The cobras’ mouths were opened using a sterile mouth gag. Two oropharyngeal swabs were collected using commercial sterile cotton-tipped swab sticks. We used the same conditions for the cultivation and isolation of these bacteria from both the wound and oropharynx [16]. Briefly, swabs were taken by rotating the cotton tip on the floor of the oral cavity and then inoculated on cetrimide agar, eosin methylene blue agar, and thiosulfate-citrate-bile salts-sucrose agar. The spread plates were incubated aerobically for 24–48 h at 37°C. Isolated pre-treatments before sampling strains were initially identified by colony morphology and gram characteristics. Subsequently, Sanger sequencing of the 16S ribosomal RNA gene was performed to confirm the identification of species.

### DNA extraction and construction of a 16S rDNA metagenomic library for *N*. *atra* oropharynx samples

Genomic DNA was extracted from each sample using the QIAamp DNA mini kit based on the manufacturer’s instructions. The Illumina 16S library preparation protocol and two-step polymerase chain reaction (PCR) were followed. The V3–V4 hypervariable region (500–600 bp) of the 16S rDNA gene segment was amplified using barcoded PCR: 16s_illumina_V3F 5′-CCTACGGGNGGCWGCAG-3′ and 16s_illumina_V4R 5′-GACTACHVGGGTATCTAATCC-3′. PCR products were purified on 0.8% agarose gel at 80 V for 40 min. Then they were quantified using the Qubit dsDNA HS assay kit (Invitrogen, Carlsbad, CA, USA) according to the manufacturer’s protocol.

### Illumina MiSeq sequencing

Purified amplicons were pooled together and paired-end sequenced at 2 × 300 bp on an Illumina MiSeq platform (Illumina, San Diego, CA, USA).

### Analysis of 16S metagenomics

Taxonomic classification was performed using the Illumina BaseSpace (v1.0.1) 16S metagenomics workflow. The Illumina-curated version of Greengenes released in May 2013 was used as the reference database. The algorithm is an implementation of the Naïve Bayesian Classifier (The Ribosomal Database Project) described by Wang Q [18].

## Results

### Clinical characteristics of *N*. *atra* bite patients

Positive deep tissue or biopsy microbial cultures were identified in 15 (nine men (60%) and six women (40%)) of 86 total cobra bite patients. The patients’ median age was 54 years (interquartile range, IQR: 38–66 years). The bite site occurred on the upper limb in six cases (40%) and the lower limb in eight cases (53.3%). The other case was bitten on the right side of the neck while in bed. Local effects such as suspected acute compartment syndrome, skin necrosis, bullae or blisters, local numbness, necrotizing adiposities, and necrotizing fasciitis were observed in 2, 15, 5, 5, 3, and 12 patients, respectively. Systemic effects such as fever (≥38°C), rhabdomyolysis, and gastrointestinal issues were observed in 12, 2, and 7 patients, respectively. The median dose of bivalent antivenom for *N*. *atra* and *B*. *multicinctus* administration was 15 vials (IQR: 12–16). The elapsed times between the bite and the first dose of antivenom administration were <6, 6–12, and >12 h in 11, 1, and 3 patients, respectively. The surgical indication was wound necrosis with secondary infection for all 15 patients. Cleansing procedures included fasciotomy or fasciectomy, debridement, and finger or toe amputation in 9, 12, and 1 patient, respectively. Reconstructive procedures comprised a split-thickness skin graft or full-thickness skin graft and flap surgery in 12 and 7 patients, respectively. The median elapsed time between the bite and the first operation was 3 days (IQR, 1–4 days). The median elapsed time between the bite and obtaining deep tissue or a biopsy culture was 4 days (IQR, 2–8 days). Antibiotics were administered to all patients upon arrival at the emergency department before obtaining a deep tissue or biopsy culture, and they included penicillin, oxacillin, amoxicillin/clavulanic acid, ampicillin/sulbactam, cefazolin, gentamicin, and metronidazole in 1, 3, 2, 6, 12, 8, and 2 patients, respectively (Table 1).

**Table 1:** Demographic data and management of patients with positive deep tissue or biopsy culture.

Demographic data	Case number, n = 15
Age (median, IQR)	54 (38–66)
Male (%)	9 (60)
Body part of bitten	
Upper limb	6
Lower limb	8
Trunk/Others	1 (neck)
**Local complications**	
Tissue swelling^a^	
Minimal	0
Mild	1
Moderate	8
Severe	5
Acute compartment syndrome, suspected	2
Skin necrosis	15
Bullae/blister	5
Local numbness	5
Lymphangitis/lymphadenitis	0
Necrotizing soft tissue infection	
Necrotizing adiposities	3
Necrotizing fasciitis	12
Finger or toe gangrene	0
**Systemic complications**	
Fever (≥38°C)	12
Rhabdomyolysis^b^	2
Gastrointestinal effect^c^	7
Ptosis or muscle weakness	0
**Management**	
Time elapsed between bite and first dose of antivenom^d^ in h	
<6 h	11
6–12 h	1
>12 h	3
Total antivenom dose in vial (median, IQR)	15 (12–16)
Operation case	
Fasciotomy/fasciectomy	9
Debridement	12
STSG/FTSG^e^	12
Flap	7
Finger or toe amputation	1
Time elapsed between bite and first operation in days (median, IQR)	3 (1–4)
Time elapsed between bite and obtaining deep tissue or biopsy culture in days (median, IQR)	4 (2–8)
**Antibiotics administered prior to obtaining deep tissue or biopsy culture 15**	
Penicillin	1
Oxacillin	3
Amoxicillin/clavulanic acid	2
Ampicillin/sulbactam	6
Cefazolin	12
Gentamicin	8
Metronidazole	2

^a^: minimal: local swelling at the bite site; mild: swelling involving a whole hand or foot; moderate: swelling from the hand to the forearm, or from the foot to the leg; severe: swelling extending to the arm, thigh, or the above area, from a hand or foot bite. Because Blaylock’s classification cannot be applied to bites on the body or trunk, the patient bitten on the neck was not included in the swelling grade analysis despite serious tissue swelling;

^b^: creatine kinase level of 2599 and 4306 U/L, respectively;

^c^: including nausea, vomiting, abdominal upset, or diarrhea;

^d^: bivalent specific antivenom for *Naja atra* and *Bungarus multicinctus*;

^e^: split-thickness skin graft and full-thickness skin graft.

### Identification of bacteria from infected *N*. *atra* bite wounds based on the VITEK 2 system

Nine bacteria were isolated from infected cobra bite wounds (Table 2). *Enterococcus* sp. (aerobic gram-positive bacteria) was present in seven cases and was the most common gram-positive pathogen. The most common aerobic gram-negative bacterial species was *Morganella morganii* (10 cases). *Proteus vulgaris* and *Serratia marcescens* were isolated from two cases. *P*. *mirabilis*, *P*. *penneri*, *Providencia rettgeri*, *Pseudomonas aeruginosa*, and *Shewanella* sp. were found in one case each. In addition, 10 patients had wounds with at least two bacterial pathogens. Simultaneously, an anaerobic culture was performed in 11 of the 15 patients, but no anaerobic bacteria were found in the study.

**Table 2:** Bacteria isolated from infected cobra bite wound culture.

Species	Case number
**Aerobic gram-positive bacteria**	
*Enterococcus* sp.	7
**Aerobic gram-negative bacteria**	
*Morganella morganii*	10
*Proteus mirabilis*	1
*Proteus penneri*	1
*Proteus vulgaris*	2
*Providentia rettgeri*	1
*Pseudomonas aeruginosa*	1
*Serratia marcescens*	2
*Shewanella* sp.	1
**polymicrobial: ≥ 2 pathogens**	10

### Identification of bacteria from the *N*. *atra* oropharynx through conventional microbial culture-based 16S Sanger sequencing

Because *N*. *atra* is rarely found, we collected five adult snakes for the experiment. After obtaining swabs from their oropharynx, 54 single colonies were recovered on a culture plate. We then identified 18 bacterial species using the 16S Sanger sequencing method (S1 Table). The colonies consisted of *M*. *morganii* in 17 clones (31.5%), followed by *Bordetella petrii* in 6 clones (11.1%) and *Corynebacterium freneyi* in 5 clones (9.3%). Therefore, *M*. *morganii* was the principal bacterium isolated from both cobra bite wounds and the cobra oropharynx (Tables 2 and S1).

### A comparison between bacterial microbiota in *N*. *atra* bite wounds and the cobra oropharynx

**Fig 1:**
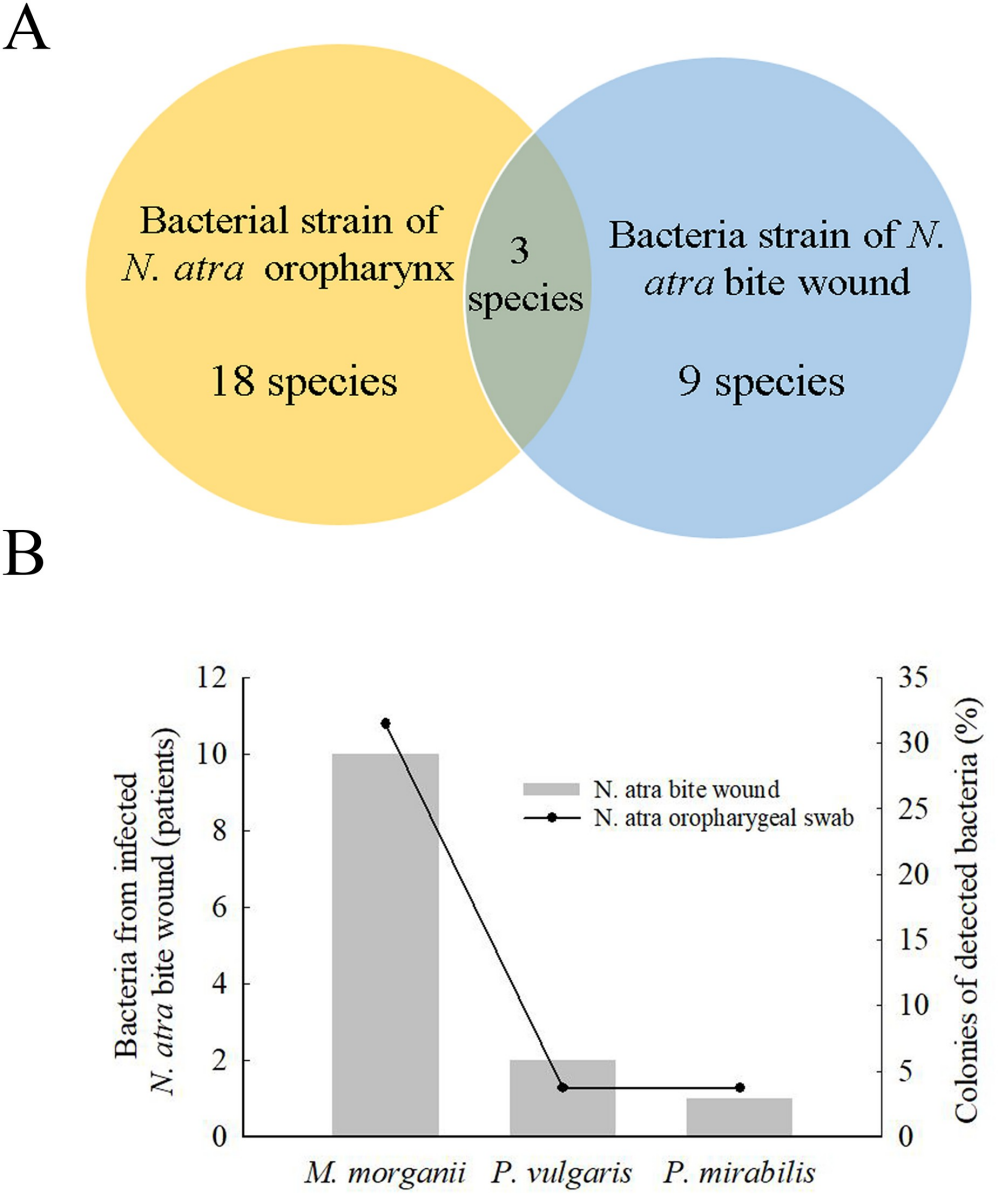
Bacteria in *N*. *atra* bite wounds and cobra oropharyngeal swabs. (A) Venn diagram indicates that 18 bacterial species strains (yellow) were detected in the oropharynx of *N*. *atra* by culture-based method, and 9 species were detected from bite wounds (blue) by using the VITEK 2 system. Three bacteria were both present in the oropharynx of snake and the bite wounds. (B) The most commonly species were *Morganella morganii*, *Proteus vulgaris*, and *Proteus mirabilis*.

To understand the microbial diversity of snakebite wounds and identify the relative proportion and types of species in the wound, we compared cultured bacteria between cobra bite wounds and the cobra oropharynx. Among bacterial strains, 18 species were isolated from the cobra oropharynx using 16S Sanger sequencing, and 9 species were isolated from cobra bite wounds using the VITEK 2 system (Fig 1A). Overall, the microbial culture method identified only three bacterial species (i.e., *M*. *morganii*, *P*. *vulgaris*, and *P*. *mirabilis*) in both cobra bite wounds and the oropharynx of cobras (Fig 1A), and these three bacterial species accounted for 31.5%, 3.7%, and 3.7% of those in the cobra oropharynx, respectively (Fig 1B, right vertical bar), and appeared in the bite wounds of 10, 2, and 1 patients, respectively (Fig 1B, left vertical bar).

### Identification of bacterial microbiota in the cobra oropharynx through next-generation sequencing

To better understand the bacterial microbiota in the cobra oropharynx, oropharynx swabs from five cobras were collected, and 16S metagenomic sequencing was performed. To model oropharynx bacterial microbiota, we calculated the number of identified species using operational taxonomic units (OTUs) and the abundance of species using the Shannon species diversity index. The OTU range and the Shannon species diversity index were 286–416 and 1.907–2.224 in the cobra oropharynx, respectively (Table 3). Taxonomic classification was conducted using the Ribosomal Database Project Classifier and Greengenes Database. The five most abundant bacterial species accounted for 84% ± 2.9% of those in the oropharynx bacterial microbiota. The 16S metagenomic sequencing data revealed that the most common bacterial species in the oropharynx of all five cobras were *P*. *azotoformans*, *P*. *lundensis*, *Delftia tsuruhatensis*, and *Methylobacterium goesingense*. These species accounted for 27.7% ± 8.7%, 22.3% ± 11.6%, 20.3% ± 1.9%, and 9.0% ± 1.7% of the oropharynx bacterial microbiota, respectively. In addition, through the NGS method, we found that the only four bacteria present in both *N*. *atra* bite wounds and the oropharynx were *Enterococcus* sp., *Morganella morganii*, *Proteus mirabilis*, and *Proteus penneri* (Table 4).

**Table 3:** Bacteria identified in the oropharynx of *N*. *atra* based on the 16S metagenomics method.

Sample ID	Shannon Species Diversity	Number of Species Identified (OTU)	Top 5 species				
			1	2	3	4	5
**MS18121-1**	1.907	404	*Pseudomonas azotoformans*	*Pseudomonas lundensis*	*Delftia tsuruhatensis*	*Methylobacterium goesingense*	*Delftia lacustris*
			29.82%	22.44%	20.73%	9.21%	2.78%
**MS18121-2**	2.224	362	*Pseudomonas azotoformans*	*Pseudomonas lundensis*	*Delftia tsuruhatensis*	*Methylobacterium goesingense*	*Kushneria indalinina*
			25.55%	20.65%	18.65%	8.71%	6.46%
**MS18121-3**	2.065	411	*Pseudomonas lundensis*	*Delftia tsuruhatensis*	*Pseudomonas azotoformans*	*Methylobacterium goesingense*	*Dysgonomonas wimpennyi*
			39.58%	18.09%	14.52%	6.36%	2.59%
**MS18121-4**	2.015	286	*Pseudomonas azotoformans*	*Delftia tsuruhatensis*	*Methylobacterium goesingense*	*Pseudomonas fluorescens*	*Pseudomonas lundensis*
			38.12%	22.33%	11.06%	8.84%	7.06%
**MS18121-5**	1.998	416	*Pseudomonas azotoformans*	*Delftia tsuruhatensis*	*Pseudomonas lundensis*	*Methylobacterium goesingense*	*Delftia lacustris*
			30.52%	21.67%	21.52%	9.75%	2.99%

**Table 4:** Identification of the bacterial species in N. atra oropharynx and bit wounds.

Species	16S Metagenomics NGS (oropharynx)	Sanger sequencing of bacterial culture (oropharynx)	Vitek2 system of bacterial culture (bite wounds)
*Stenotrophomonas maltophilia*	O	X	X
*Acinetobacter baumannii*	O	X	X
*Morganella morganii*	O	O	O
*Proteus mirabilis*	O	O	O
*Proteus penneri*	O	X	O
*Enterococcus spp*.	O	O	O

### Comparison of bacterial microbiota in *N*. *atra* oropharynx identified through 16S Sanger sequencing of bacterial culture and 16S metagenomic sequencing

**Fig 2:**
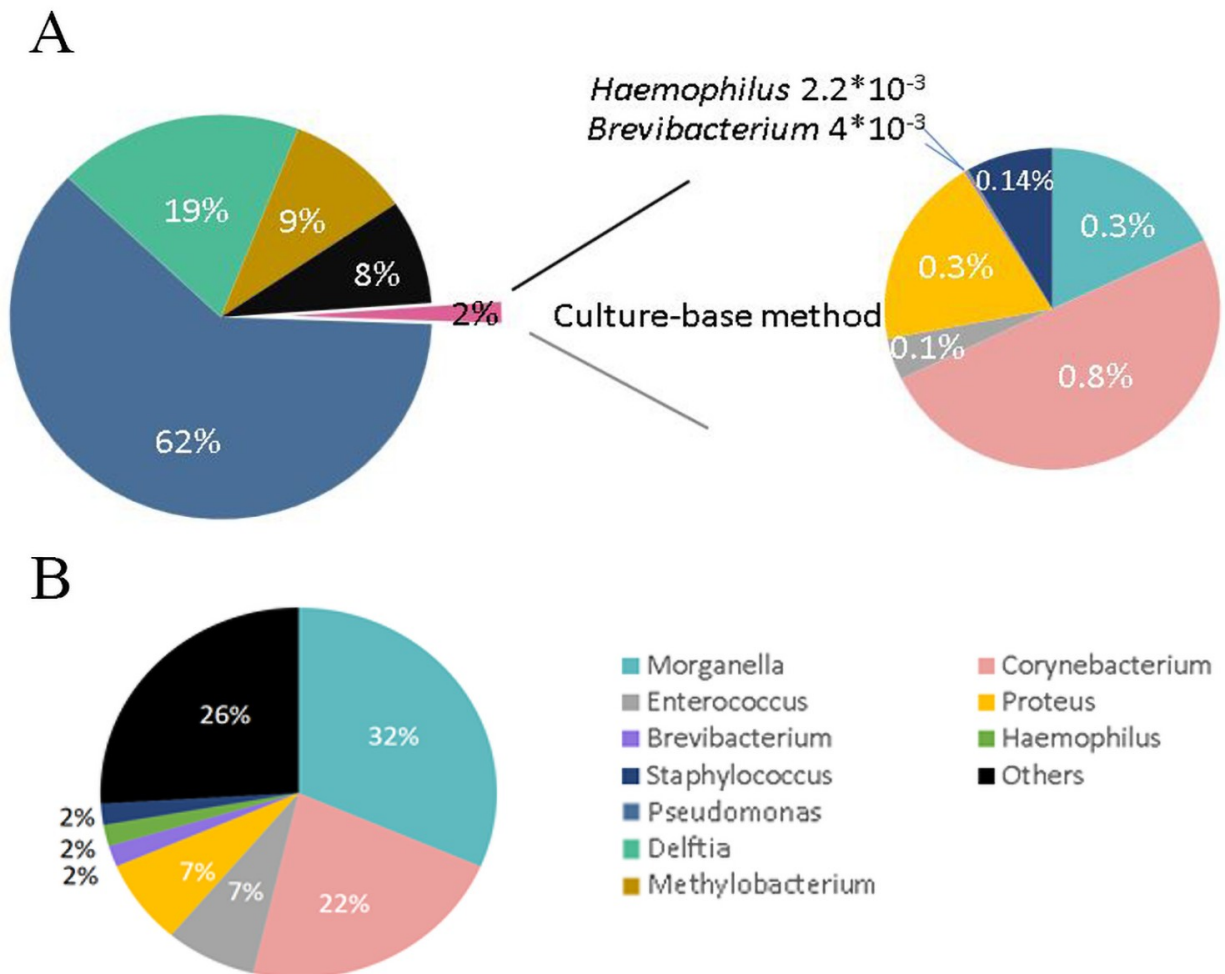
Pie charts of the abundance of 16S bacteria from the oropharynx of *N*. *atra* identified through next-generation sequencing (NGS) and culturing (genus level). (A) Bacterial abundance identified through the swabs of the oropharynx of *N*. *atra* and 16S rDNA through NGS (left). The culture-based method only detected 2% of all bacteria uncovering by NGS method (right). (B) The proportion of detected bacteria at the genus level through culturing.

We grouped the bacteria by genus to compare the bacterial microbiota of the cobra oropharynx identified through 16S Sanger and metagenomic sequencing. The 16S metagenomic sequencing data indicated that the most representative bacterial genera were *Pseudomonas* (62%), *Delftia* (19%), and *Methylobacterium* (9%), but none of these genera were detected in the bacterial culture (Fig 2A, left). By contrast, the most representative genera, as obtained from the cultured clones, were *Morganella* in 31.5% of the clones, *Corynebacterium* in 22.2%, *Proteus* in 7.4%, and *Enterococcus* in 7.4% (Fig 2B). Only 2% of the bacteria identified through 16S metagenomic sequencing were found by Sanger sequencing of the bacterial culture (Fig 2A, right). The proportions of each genus in 2% of the bacteria were identical in these two methods, i.e., 0.83%, 0.3%, and 0.32% for the genera *Corynebacterium*, *Morganella*, and *Proteus*, respectively (Fig 2A, right). Notably, the pathogenic bacteria *Stenotrophomonas maltophilia* and *Acinetobacter baumannii* were identified by 16S metagenomic sequencing, which were undetected in both wounds using the VITEK 2 system and the cobra oropharynx using 16S Sanger sequencing of the microbial culture (Table 4).

## Discussion

*N*. *atra* belongs to the family Elapidae, but its bite causes minimal or insignificant neurotoxicity. Furthermore, necrotizing soft tissue infection was the principal manifestation of its bite [5, 19]. We demonstrated that the use of empiric antibiotics in *N*. *atra* bite management was probably widespread but may not have been directly effective on pathogens. The most common pathogen recovered from deep tissue or biopsy culture after wound debridement was *M*. *morganii*. This bacterium is naturally resistant to various antibiotics, including penicillin, amoxicillin, ampicillin, oxacillin, cefazolin, and sulfamethoxazole [20]. In Taiwan, Chen et al. suggested that amoxicillin/clavulanate with ciprofloxacin or piperacillin/tazobactam is the antibiotics of choice in the management of snakebites because *Enterococcus* spp. and *M*. *morganii* are frequently present in the infected bite wound [21]. Mao et al. and Chiang et al. suggested that first-generation cephalosporin with aminoglycoside or sulfamethoxazole/trimethoprim is the preferable antibiotics in the management of infected *Protobothrops mucrosquamatus* and *Trimeresurus stejnegeri stejnegeri* bites [22, 23]. These suggestions are based on conventional wound microbial culture without prior determination of antibiotic administration before obtaining cultures, thus rendering these suggestions biased.

In Thailand, Theakston et al. recommended benzylpenicillin with gentamicin as a prophylactic antibiotic regimen after *Calloselasma rhodostoma* envenomation because *Enterobacter*, *Pseudomonas*, *Staphylococcus*, and *Clostridium* spp. were cultured from the venom and mouth of this snake [24]. In Brazil, Jorge et al. suggested the use of chloramphenicol as the antibiotic of choice for managing *Bothrops* envenomation because the most frequently isolated pathogens from these wounds include *M*. *morganii*, *Providencia rettgeri*, *Enterobacter* sp., *Escherichia coli*, *Enterococcus* sp., and *Bacteroides* sp. [25]. However, in Ecuador, a controlled study suggests that chloramphenicol and gentamicin were ineffective in preventing abscess formation (9 cases among 114 snake bites) given that they are administered for 24 h and less than half of the patients are identified as *Bothrops* bites [26]. In the United States, prophylactic antibiotic administration is not recommended for treating rattlesnake bites given the low incidence of wound infection (0.98%) [27]. Although the administration of specific antivenom remains the standard of care in the management of snakebites, administration of prophylactic, empiric antibiotics, or both is controversial. This is not only because the diagnosis of secondary infection is confounded by the venom’s effects but also because the microbiology of the infected wound and oropharynx of the culprit snake is not well established [7]. In addition, a recent study demonstrated that the bivalent antivenom for *N*. *atra* and *B*. *multicinctus* is ineffective against the necrotic effect of the venom [15]. Because most *N*. *atra* bite patients underwent surgery for secondary wound infections, this seems to be a role for empiric antibiotics in the management of severe local or systemic septic complications following a bite.

The snake oral cavity contains various aerobic and anaerobic bacteria, particularly fecal gram-negative rods because their prey (e.g., mammals, reptiles, and fish) usually defecate while being ingested [3, 28, 29]. The bacterial composition varies across snake species and may be influenced by venom properties [24, 30, 31] and the fecal flora of the prey in different geographic regions [29]. On the basis of conventional microbial culture methods, *Enterobacter*, *Proteus*, and *Pseudomonas* were the most frequently isolated gram-negative rods, *Enterococcus* and *Micrococcus* were the most common gram-positive cocci, and *Clostridium* was the most common anaerobic bacteria found in the fangs or venom of *Crotalus atrox* [32]. *P*. *aeruginosa*, coagulase-negative *Staphylococcus* sp., *Alcaligenes* sp., and *Enterobacter cloacae* were all isolated from the venom of *C*. *viridis helleri*, and *Bacillus* sp., *Proteus rettgeri*, and coagulase-negative *Staphylococcus* sp. were observed in *C*. *scutulatus* [29]. Furthermore, *Stenotrophomonas maltophilia*, *P*. *aeruginosa*, and *Diphtheroid bacillus* were obtained from *Trimeresurus albolabris* [13]. In contrast to crotaline snakes, the oral cavity of *N*. *atra* harbors more complex pathogenic bacteria, including gram-negative bacterial species *M*. *morganii*, *Aeromonas hydrophila*, and *Proteus*, and gram-positive bacteria, like *Enterococcus faecalis*, coagulase-negative *Staphylococcus* sp., and anaerobic species (*Clostridium*), which may be associated with higher incidences of bite wound infection (i.e., 76%–81%) [12, 13]. However, in *N*. *mossambica* bites, patients had serious wound necrosis or necrotizing soft tissue infection even with the use of specific antibiotics or third-generation cephalosporin [10]. This can be caused by additional wound necrosis-inducing bacteria that were undetected, unreported, or both in conventional microbial culture in addition to venom-induced cytotoxicity and local tissue damage.

Currently, microbial culture remains the principal method to detect bacteria in clinical practice. Meanwhile, the accuracy and reliability of NGS of 16S metagenomics have rapidly improved [33]. We used NGS to examine the bacterial microbiota of the oropharynx of *N*. *atra* and to construct a better model of a snake oropharynx microbial profile, which may have therapeutic implications. Our NGS data indicated that the bacterial microbiota, as identified using OTUs, included more than 286 species. However, using the culture-based method, only 18 bacterial species could be identified in the same sample from the cobra oropharynx and only 9 species in infected wounds. The lower number of bacterial species identified in deep tissue or biopsy culture in this study compared with the Mao et al. study is probably due to the lower number of bacteria in deep tissue or from biopsy compared with wound swab sampling, fastidious or uncultivable bacteria, antibiotic therapy, or several of these aspects initiated before sampling [7, 34]. Only three bacterial species recovered from deep tissue or biopsy cultures were also present in the oropharynx swab culture of *N*. *atra*, which suggests the requirement of simultaneous determination of both the bite wound and snake oropharynx bacterial microbiota. Given the considerable variability in the incidence of infection in snake bite wounds (1%–81%) [35, 36], the causative organisms, and the methods used to isolate a pathogen in variable clinical settings [5, 10, 27, 37–39], an institutional protocol for antibiotic selection during snake bite wound management should be adapted based on the regional microbiology data of both snake bite wounds and snakes.

Rampini et al. suggested that the 16S rRNA gene sequencing method identifies many more culture-negative bacteria compared with microbial culture [40]. *Pseudomonas* was the most abundant genus found in the cobra oropharynx cavity. In particular, *P*. *azotoformans* is an environmental bacterium that can be isolated from soil and plants. In 2020, a bacterial isolate from rabbit meat samples was treated with blue and fluorescent pigments, and the growth of uniform bacterial colonies that were visible through fluorescent pigmentation indicated *P*. *azotoformans*, which belongs to the fluorescent group [41]. *P*. *lundensis* is a new species of the *Pseudomonas* genus and can produce fluorescent pigments and catalase and grow at 0°C. Furthermore, they are spoilage and pathogenic bacteria found in refrigerated meat [42]. *D*. *tsuruhatensis*, which was initially considered an environmental bacterium, was later confirmed as a human pathogen [43]. In addition, it can be misidentified as a closely related nonpathogenic species, *D*. *acidovorans*, using the VITEK 2 system with a probability level of 98% [43]. Moreover, the pathogenic bacteria *Stenotrophomonas maltophilia* and *A*. *baumannii* detected in the *N*. *atra* oropharynx were not found in deep tissue or biopsy cultures of infected wounds. It is worth noting that *S*. *maltophilia* is a multiple-drug-resistant pathogenic bacterium that is frequently involved in pneumonia and bacteremia in humans [44].

Several reports have presented potential advantages of molecular diagnostics over microbial culture, namely, a shorter turnaround time, detection of difficult to grow bacteria, or detection after prior administration of antibiotics that inhibit bacterial growth [34]. Although PCR amplification bias and 16S rRNA copy variation may cover up real relative abundance, numerous studies have shown that the NGS method of 16S metagenomics can achieve reasonable quantification accuracy for complex microbial communities [33]. In addition, a traditional microbiology laboratory only reports a few bacteria in the culture and clinically associates it with polymicrobial infections. The advantage of the NGS method 16S metagenomics assay is not only the identification but also the quantification of the relative abundance of all bacteria in polymicrobial infections [33]. Furthermore, 16S rRNA sequencing identifies anaerobic bacteria better than the VITEK 2 system [45].

Advances in sequencing technology in the last decades have made the molecular identification of bacteria widely available, and an increasing number of studies have focused on the clinical applications of this technology. The main advantage of NGS-based sequencing is that it permits the identification of 16S rRNA on uncultured samples and provides fast and comprehensive analysis of the microbial profile, thereby reducing the time required for clinical diagnosis and treatment. However, we are in the early stages of understanding the microbiome of snakes and snakebite wounds. Generally, bacterial isolates from the oral cavity of snakes are not all pathogenic: they may cause wound infections or abscesses in humans but so do normal environmental contaminants or soil pathogens [46]. Although bacterial pathogens can make some people critically ill, many people who asymptomatically carry these organisms are often unaware that they are infected, and it is difficult to draw a line between the asymptomatic presence of such pathogens and the normal microflora [47]. We do not know if antibiotic treatment must be directed at each isolated organism, at only presumed bacterial ringleaders, or even at organisms that were once considered probably nonpathogenic lab weeds [46]. Nevertheless, ascertaining this information should be the initial stage of any study that attempts to establish appropriate antimicrobial therapy for *N*. *atra* envenomation.

### Limitations

There are several limitations in our study. First, there was usually a delay of several days between the bite and the debridement surgery (to allow demarcation of the necrosis). Empiric antibiotic administration for wound infection and before obtaining microbial cultures during debridement surgery might have changed the bacterial microbiota of the wound. However, owing to the high incidence of wound infection following an *N*. *atra* bite [5, 7], it is not possible or even ethical to postpone the antibiotic administration while waiting for the culture report. Although the clinical data in 2001–2006 were analyzed, no optimized protocol is currently established in the management of *N*. *atra* bites because the diversity of pathogens might have been underestimated in the clinical setting based on conventional culture techniques and various pretreatments before sampling. Second, we did not submit the deep or biopsy tissues for 16S Sanger sequencing or NGS evaluation because the techniques are not available during the patients’ enrollment period and *N*. *atra* bites are rare in Taiwan. Third, only five adult *N*. *atra* were analyzed, and hence, these data may not be properly representative of the wild population of this snake. In addition, the changing patterns of bacterial microbiota in snakebite wounds and the *N*. *atra* oropharynx throughout the year remain unknown, although numerous studies reported the increasing resistance of various bacteria [48, 49]. Nevertheless, this is the first study to investigate the bacterial microbiota of the oropharynx of *N*. *atra* with utilization of NGS and Sanger sequencing methods. Our study findings may have therapeutic implications for *N*. *atra* bite wound infections and shed light on the complex mechanisms of wound necrosis following a bite.

## Supporting information

S1 Table Bacteria identified by Sanger sequencing of 16S ribosomal RNA gene method. (DOCX)
