# Supplementary material for: An investigation of conventional microbial culture for the Naja atra bite wound, and the comparison between culture-based 16S Sanger sequencing and 16S metagenomics of the snake oropharyngeal bacterial microbiota
Source: PLoS Negl Trop Dis. 2021 Apr 15;15(4):e0009331. doi: 10.1371/journal.pntd.0009331 (PMC8078740; doi:10.1371/journal.pntd.0009331)
Supplement: S1 Table — (DOCX) [file pntd.0009331.s001.docx]

**S1 Table. Bacteria identified by Sanger sequencing of 16S ribosomal RNA gene method.**

| Species | Ident. | Accession | Colony counts |
| --- | --- | --- | --- |
| Morganella morganii | 99 | LR133904.1 | 17 |
| Bordetella petrii | 100 | MK629823.1 | 6 |
| Corynebacterium freneyi | 99 | EF462406.1 | 5 |
| Enterococcus faecalis | 100 | MK894863.1 | 4 |
| Corynebacterium auriscanis | 95 | EU911931.2 | 3 |
| Uncultured bacterium clone ncd542d11c1 | 99 | HM277642.1 | 3 |
| Corynebacterium sphenisci | 94 | CP009248.1 | 2 |
| Proteus mirabilis | 100 | MH985194.1 | 2 |
| Proteus vulgaris | 98 | MG438540.1 | 2 |
| Xanthomonadaceae bacterium | 100 | KF322147.1 | 2 |
| Brevibacterium sp. 7400794 | 99 | GQ222264.1 | 1 |
| Corynebacterium lactis | 95 | LT991961.1 | 1 |
| Corynebacterium nasicanis | 98 | NR_145583.1 | 1 |
| Haemophilus influenzae | 90 | JN227863.1 | 1 |
| Pasteurellaceae bacterium | 90 | EU826036.1 | 1 |
| Staphylococcus aureus | 100 | MK780055.1 | 1 |
| Uncultured Corynebacterium sp. clone T1909 | 99 | HQ616240.1 | 1 |
| Uncultured Morganella sp. clone F3C6 | 100 | KF945097.1 | 1 |
